# Long-Term Performance of Biofilters Packed with Manure-Derived Media for Trimethylamine Removal: Operational Responses and Microbial Succession

**DOI:** 10.3390/ani16213420

**Published:** 2026-10-29

**Authors:** Ketian Chen, Le Liu, Qiang Zhang, Zhiqi Dai, Lu Luo, Chunmei Li

**Affiliations:** 1 Research Center for Livestock Environmental Control and Smart Production, College of Animal Science and Technology, Nanjing Agriculture University, Nanjing 210095, China; ketianchen@stu.njau.edu.cn (K.C.); lle@stu.njau.edu.cn (L.L.); 2022805114@stu.njau.edu.cn (Z.D.); 2 Department of Biosystems Engineering, University of Manitoba, Winnipeg, MB R3T 5V6, Canada; qiang.zhang@umanitoba.ca

**Keywords:** biofilter, odor, trimethylamine, filter media, microbial community

## Abstract

Four laboratory-scale biofilters were designed and evaluated for treating trimethylamine (TMA). Each biofilter was measured as being 68 cm in height and 15 cm in diameter. The filter media consisted of various mixtures of dairy manure compost, dairy barn bedding, crushed corn cobs, wood chips, and sawdust. This study aimed to compare the removal efficiencies of TMA achieved by biofilters packed with different media compositions, as well as to analyze the succession patterns in the structure of microbial communities on the media surfaces during operation. Contaminated air streams were artificially generated by bubbling air through a TMA solution, resulting in inlet concentrations ranging from 1.88 to 4.23 mg/m^3^. Under empty bed residence times (EBRTs) ranging from 42.7 to 28.0 s, all four biofilters achieved removal efficiencies exceeding 60%. However, the performance varied among the biofilters. The biofilter packed with a mixture of matured compost, wood chips, and crushed corn cobs at a volume ratio of 10:9:1 exhibited the highest removal efficiency (>83%). Ammonia was detected at the outlet of the biofilters as a byproduct of microbial TMA degradation, accompanied by the accumulation of ammonium nitrogen in the filter media. Microbial community analysis of the high-performance biofilter revealed a progressive increase in microbial complexity and diversity over 95 days of operation. *Glycomyces* was the dominant genus throughout the operation and showed a significant positive association with TMA removal efficiency, but its specific role in TMA degradation remains to be experimentally confirmed.

## Simple Summary

Livestock farming releases substantial amounts of trimethylamine (TMA), a typical nitrogenous odor gas that can adversely affect regional air quality and the environmental conditions of livestock housing, with potential implications for animal health and welfare. Biofiltration is a low-cost and environmentally friendly odor control technology, yet the long-term stability and application potential of TMA removal by composite biofilter packing materials remain unclear. In this work, four groups of composite biofilters based on common agricultural and livestock waste substrates were operated for a 95-day continuous long-term experiment to explore their TMA purification performance under different operating conditions. The results showed that all composite packing materials achieved stable TMA purification effects, with the optimal substrate ratio maintaining a TMA removal efficiency of over 80%. Inlet pollutant concentration and gas residence time were key factors affecting biofilter treatment performance. Moreover, nitrogen accumulation and secondary ammonia emission occurred during biofiltration, accompanied by an obvious succession in bacterial community diversity in the optimal biofilter system. This study verifies that low-cost manure- and crop waste-based composite fillers can be reliably applied to long-term TMA odor purification, and it clarifies the correlation between operational conditions, microbial variation and treatment efficiency. It provides practical support and theoretical reference for the optimization and engineering application of livestock odor biofiltration systems.

## 1. Introduction

In recent years, dairy production has increasingly shifted toward large-scale, intensive farming systems, improving production efficiency while also intensifying environmental challenges associated with malodorous emissions from livestock housing and manure management. These emissions can deteriorate air quality within and around livestock facilities and may adversely affect the environmental conditions experienced by both farm workers and animals. Previous research by our research team found that the mean concentration of trimethylamine (TMA) measured in a low-profile, cross-ventilated dairy barn was 1.08 mg/m^3^. Although TMA was present at lower concentrations than dimethyl sulfide and dimethyl disulfide, its comparatively low odor threshold suggests that it may still make a substantial contribution to the overall odor burden. TMA is primarily generated through the microbial degradation of carnitine, choline, betaine, and related compounds [1]. In dairy production systems, the interaction between feces and urine represents an important source of TMA emissions. Sintermann et al. [2] reported substantial quantities of TMA and its precursors in dairy cattle excreta, noting that trimethylamine oxide (TMAO) in manure and urine can be rapidly converted to TMA by microbial activity under typical barn conditions, with an average emission rate of approximately 0.6 μg/m^2^·s. Previous studies have demonstrated that TMA inhibits the synthesis of biological macromolecules, including DNA, RNA and proteins, and may exert teratogenic effects on animal embryos [3]. Furthermore, TMA not only causes distress to the respiratory tract but may also react with nitrites to form highly carcinogenic nitrosamines, presenting serious health hazards [4]. Therefore, effective control of TMA emissions is important for odor management and for improving air quality in livestock housing.

Various technologies have been developed for mitigating malodorous gases and can be broadly classified into physical, chemical, and biological deodorization methods [5,6]. Among these, biological deodorization is an attractive option because of its relatively low operating costs, simple operation and maintenance, and potential for the simultaneous removal of multiple gaseous pollutants [7,8]. Three major configurations of biological odor treatment are biofilters (BFs), biotrickling filters (BTFs), and bioscrubbers (BSs). In BFs, contaminated gas passes through a porous packing bed colonized by microorganisms, whereas BTFs employ irrigated packing materials that support microbial biofilms [9]. In BSs, gaseous pollutants are first transferred to a liquid phase and subsequently biodegraded in a separate bioreactor. Among available biological deodorization technologies, BFs stand out as an effective and environmentally benign option [10,11]. It offers multiple advantages, including coordinated abatement of diverse gaseous pollutants, potential for nitrogen recovery, low operational cost, operational simplicity, and high stability [12,13]. In biofiltration, TMA is transferred from the gas phase to the moist packing medium, where it is metabolized by microorganisms. Bacterial degradation of TMA can proceed via two pathways [14,15]. Under aerobic conditions, TMA is first oxidized to trimethylamine N-oxide (TMAO) by trimethylamine monooxygenase (Tmm). TMAO is subsequently demethylated to dimethylamine (DMA) by TMAO demethylase, and DMA is further converted to methylamine by dimethylamine monooxygenase. Methylamine can then be further degraded to formaldehyde and ammonia by methylamine monooxygenase. Under anaerobic denitrifying conditions, TMA is sequentially demethylated to DMA and methylamine through the activities of trimethylamine dehydrogenase (TMADH) and dimethylamine dehydrogenase, respectively. Both aerobic and anaerobic TMA degradation pathways have been reported in *Paracoccus* sp. Strain T231, in which Tmm and TMADH initiate TMA degradation under aerobic and anaerobic denitrifying conditions, respectively [16]. In another study, a BTF inoculated with *A. aminovorans* showed removal efficiencies higher than 98% in a range of loading rates of 0.2 to 8 m^−3^·h^−1^ at EBRT of 85 and 180 s [17,18]. However, the occurrence and relative contributions of these pathways in manure-based biofilters remain to be experimentally verified.

The filter medium is a critical determinant of both removal efficiency and long-term operational stability of biofilters [19,20]. Typically, the filter medium is a porous material with a large specific surface area and strong moisture-retention capacity, which supports microbial colonization and activity. In this study, mature compost and recycled cattle-manure-based bedding were selected as organic substrates to provide nutrients and support microbial growth, while sawdust and crushed corn cobs were incorporated as lignocellulosic bulking materials to maintain bed porosity, facilitate gas flow, reduce the risk of compaction, and provide a slowly degradable carbon source. In agricultural applications, various waste-derived materials, such as sawdust, straw and manure compost, have been investigated as biofilter media. For example, Fedrizzi et al. [21] evaluated different combinations of compost, sawdust, straw, manure compost, and wood chips in biofilters for methane removal in dairy barns and found that the filter performance was influenced by media acclimation and temperature. Zhong et al. [20] reported that the degree of manure pre-composting affected ammonia removal and nitrogen loss. However, comparative studies focusing on manure-derived filter media as primary substrates remain limited, particularly the in-depth investigation of microbial community succession associated with different substrates. Therefore, this study aimed to (i) evaluate the long-term TMA removal performance of four biofilters packed with different manure-derived media combinations; (ii) characterize their responses to changes in inlet TMA concentration, gas flow rate, and EBRT; and (iii) investigate temporal succession of the microbial community in the best-performing biofilter and explore associations between microbial taxa and biofilter performance indicators. The results of this study are intended to provide preliminary empirical insights to support the future development and evaluation of low-cost manure-derived packing materials for TMA biofiltration.

## 2. Materials and Methods

### 2.1. Biofilter Media

**Figure 1:**
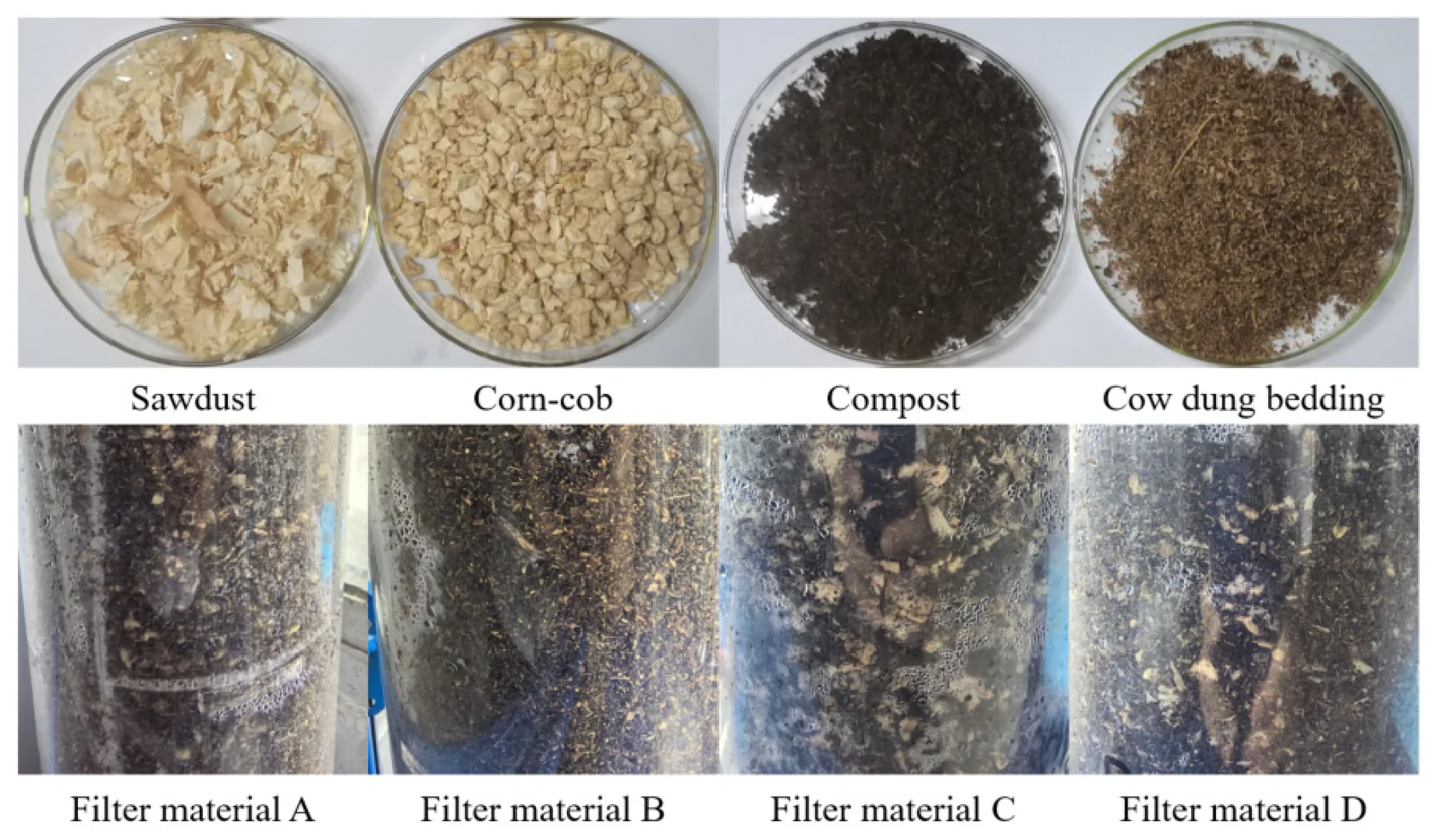
Four substrate materials, namely sawdust, chopped corn cob, cow manure compost, and cow bedding, were mixed in different ratios to prepare four different biofilter media (designated as A, B, C, and D).

Four materials, namely sawdust, crushed corn cob, cow manure compost, and cow bedding, were mixed in different ratios to prepare four different biofilter media (Figure 1). The matured compost and bedding material were obtained from two dairy farms. The bedding material was produced through a dewatering and high-temperature aerobic fermentation process. Sawdust and corn cobs were purchased from online stores.

Media A comprised cattle manure-based bedding, sawdust, and crushed corn cobs at a volume ratio of 10:9:1. Media B comprised mature compost and cattle manure-based bedding at a volume ratio of 1:1. Media C comprised mature compost, sawdust, and crushed corn cobs at a volume ratio of 10:9:1. Media D comprised mature compost, cattle manure-based bedding, sawdust, and crushed corn cobs at a volume ratio of 6:6:7:1. The formulations of filter media were established based on conventional biofilter packing strategies and subsequently optimized. A previously reported 1:1 compost-to-auxiliary-material ratio associated with effective VOC removal [22] was used as the basis for the formulations of media B and C. For medium D, compost and cattle manure-based bedding were also combined at a 1:1 ratio, with an additional 40% structural materials. In all treatments, compost or cattle manure bedding was maintained as the predominant fraction (>50%) to ensure adequate microbial activity, nutrient availability, moisture retention, and buffering capacity. Sawdust and crushed corn cobs were incorporated as structural amendments to improve porosity, reduce compaction, and promote air and moisture distribution.

Each biofilter column was constructed from acrylic glass and had an internal diameter of 15 cm, a total height of 68 cm, and a media packing height of 60 cm, corresponding to a calculated packing volume of 10.6 L. Three perforated plates were installed at heights of 4, 24, and 44 cm above the bottom of the column to support the packed bed and promote uniform gas distribution. Before loading the biofilter media, a gravel layer approximately 3 cm thick was placed on the bottom removable screen to facilitate uniform airflow and prevent clogging. During packing, the column was gently tapped after each 5 cm increment of media addition to ensure uniform packing. After every 10 cm of filling, the media was lightly compacted with a soft rod to achieve a packing density slightly higher than its natural bulk density. The four biofilters, each packed with a different medium, were designated as BF_A, BF_B, BF_C, and BF_D, respectively.

### 2.2. Experimental Setup

**Figure 2:**
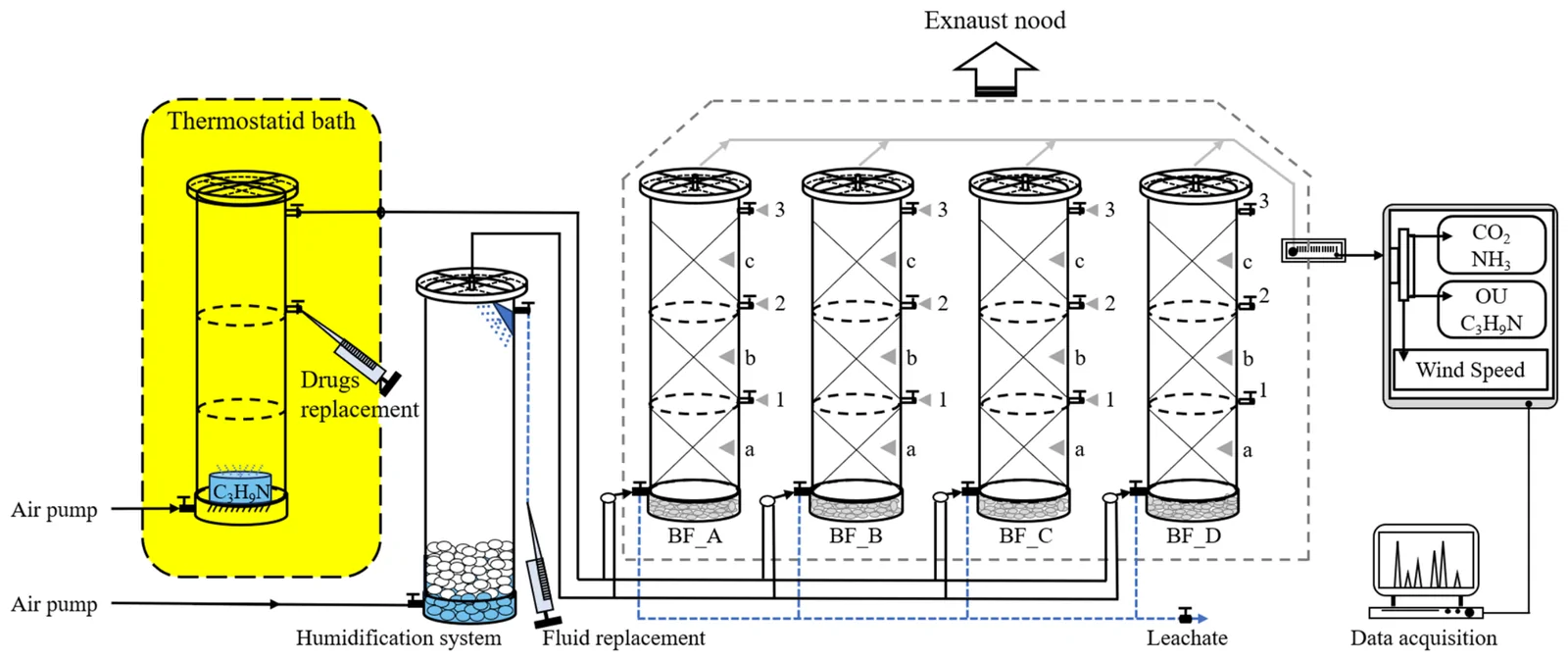
Schematic diagram of the experimental setup for testing biofilters. The different vertical layers of the biofilter packing are designated as follows: a, bottom layer; b, middle layer; and c, top layer. The numbers 1, 2, and 3 indicate the water replenishment ports located at different heights along the biofilter column.

The experimental setup consisted of a TMA vapor generator housed in a constant-temperature chamber (35 °C), a humidification column for conditioning the inlet air, four biofilters, flow-control valves for regulating airflow through each biofilter, and gas detection instruments (Figure 2). Ambient air was pumped through the TMA vapor generator to produce a TMA-containing gas stream, while a separate air pump supplied air through the humidification column to produce humidified air. The airflow through each pump was precisely controlled by adjusting the volumetric flow rates to achieve the desired TMA concentrations at the biofilter inlet. Both contaminated and clean air streams were subsequently introduced into the bottom of each biofilter via airflow control valves, which regulated the flow rate to achieve the target empty bed residence time (EBRT). An air sampling port was installed at the top of each biofilter, and the outlet gas was sampled through this port using gas detectors to measure the concentrations of CO_2_, NH_3_, and TMA (C_3_H_9_N).

Trimethylamine (TMA; CAS No. 75-50-3; 35 wt.% aqueous solution) was purchased as an analytical reagent from Anhui Zesheng Technology Co., Ltd. (Anqing, China) and used as the source of TMA vapor. Before each experiment, the stock solution was diluted with double-distilled water at a volume ratio of 1:89 (stock solution:double-distilled water, *v*/*v*), corresponding to a 90-fold volumetric dilution. Subsequently, the diluted TMA solution was dispensed into a glass beaker using a precision syringe. TMA vapor was generated by bubbling ambient air, supplied by an air pump, through the TMA solution in the beaker. Two measures were implemented to prevent desiccation of the filter media: (i) humidification of the inlet air and (ii) periodic irrigation of the filter bed. The humidification column contained deionized water and polyhedral hollow plastic beads to enhance gas–liquid contact and maintain the relative humidity of the air entering each biofilter at 85% ± 5%. Three irrigation ports, designated ports 1, 2, and 3, were installed in each biofilter to maintain the media moisture content above 40%. A volume of 50 mL of deionized water was added through each port at five-day intervals.

### 2.3. Experimental Procedure

#### 2.3.1. Biofilter Operation

The experimental period was divided into five phases: start-up phase (Phase I), stable operation phase under low inlet TMA concentration and high EBRT (Phase II), stable operation phase under high inlet TMA concentration and low EBRT (Phase III), shutdown phase (Phase IV), and reactivation phase (Phase V) (Table 1). During the first week of Phase I, the CO_2_ and NH_3_ concentrations and volumetric airflow rates at the outlet of each biofilter were measured in triplicate daily to monitor system stability. Following Phase I, the biofilters were operated continuously for 10 weeks, followed by a two-week shutdown. The biofilters were then restarted to evaluate their performance during Phase V.

The experiment was designed to evaluate biofilter performance under operational conditions representing different TMA loading regimes. During Phase I, a longer EBRT of 57.15 s was employed to facilitate microbial colonization and acclimation to the target substrate. Phase II represented relatively low inlet TMA concentration and longer EBRT, whereas Phase III combined a higher inlet TMA concentration with a shorter EBRT, resulting in a substantially different pollutant loading condition. Because inlet TMA concentration and EBRT were changed simultaneously between Phases II and III, their individual effects could not be independently separated in the present experimental design. Phase V was used to evaluate recovery of biofilter performance after a two-week shutdown period. The selected EBRT values were within the effective range reported for agricultural biofilters treating volatile amines, ensuring sufficient gas at biofilm contact time for pollutant degradation [23]. The operational duration, volumetric airflow rate, EBRT, and inlet TMA concentration for each phase are summarized in Table 1.

#### 2.3.2. Sampling of Filter Media

Filter media samples were collected from each biofilter on days 15, 42, 71, and 95 of the experiment. On each sampling date, samples were randomly collected from the bottom, middle, and top layers of the packed bed, designated as layers a, b, and c, respectively. Three subsamples were collected from each layer for physicochemical and microbial analyses. Following each sampling event, the moisture content of the remaining filter media was adjusted to approximately 55%, and additional filter medium was added to restore the original packing volume. For total nitrogen (TN) analysis, the collected samples were immediately treated with 10% (*v*/*v*) hydrochloric acid and stored at −20 °C. Samples designated for ammonium nitrogen (NH_4_^+^-N) analysis were stored at −20 °C without pretreatment. Samples for microbial community analysis were collected in sterile 5 mL tubes and stored at −80 °C.

### 2.4. Sample Analysis

#### 2.4.1. Gas Concentrations

During the trial period, TMA concentrations at the outlets of BF_A, BF_B, BF_C, and BF_D were monitored using a portable analyzer (TD600-SH-B-M9, Beijing Tiandi Shouhe Technology Development Co., Ltd., Beijing, China). NH_3_ and CO_2_ concentrations were measured using a portable multi-gas detector (GT-1000-X5, Shenzhen Corno Electronics Technology Co., Ltd., Shenzhen, China). Airflow velocity was measured using an anemometer (Testo 425, Shanghai Testo Instruments International Trading Co., Ltd., Shanghai, China). Routine measurements for biofilter performance evaluation were conducted at least three times per week during Phases II, III, and V. During Phase I, CO_2_ and NH_3_ concentrations and airflow rates were measured daily to monitor system stability, as described in Section 2.3.1, whereas Phase IV represented the shutdown period.

#### 2.4.2. Total Nitrogen (TN) in Filter Media

Acid-preserved filter media samples were oven-dried at 105 °C for 8 h until a constant mass was achieved. The dried samples were ground using a laboratory mill and passed through a 40-mesh sieve. For each sample, three analytical aliquots (approximately 0.3 g each) were weighed into glass digestion tubes. Each aliquot was digested in 10 mL of concentrated sulfuric acid in the presence of a catalyst, and TN was determined using a Kjeldahl nitrogen analyzer (Kjeltec™ 9, FOSS, Hillerød, Denmark).

#### 2.4.3. Ammonium Nitrogen (NH_4_^+^-N) in Filter Media

For NH_4_^+^-N determination, 2 g of filter media sample was extracted with 20 mL of 1 mol/L KCl solution (1:10 *w*/*v*) for 1 h. The mixture was subsequently filtered through a 0.45 μm membrane. The NH_4_^+^-N concentration in the filtrate was quantified using the Nessler reagent spectrophotometric method in accordance with the Chinese standard HJ 535-2009.

#### 2.4.4. Microbial Communities in Filter Media

Microbial community analysis based on 16S rRNA gene sequencing was performed by Shanghai Personal Biotechnology Co., Ltd. (Shanghai, China). Total genomic DNA was extracted from the filter media samples using the OMEGA Soil DNA Kit (D5635-02; Omega Bio-Tek, Norcross, GA, USA) according to the manufacturer’s protocol. The V3-V4 hypervariable region of the 16S rRNA gene was amplified using the primers 338F (5′-ACTCCTACGGGAGGCAGCA-3′) and 806R (5′-GGACTACHVGGGTWTCTAAT-3′). PCR amplification was performed under the following conditions: initial denaturation at 98 °C for 5 min; 25 cycles of denaturation at 98 °C for 30 s, annealing at 52 °C for 30 s, and extension at 72 °C for 45 s; followed by a final extension at 72 °C for 5 min. Sequencing libraries were prepared using the TruSeq Nano DNA LT Library Prep Kit and sequenced on an Illumina NextSeq platform according to the manufacturer’s protocols.

Sequencing data were processed using QIIME 2 (version 2024.5) with the DADA2 algorithm for sequence quality filtering and denoising. Paired-end reads were merged, and chimeric sequences were removed to infer amplicon sequence variants (ASVs). Taxonomic classification of each ASV was performed using the RDP Classifier with the SILVA SSU rRNA database (release 138) as the reference database and a confidence threshold of 70%. Rarefaction analysis was conducted to assess sequencing depth. Alpha diversity was assessed using the Chao1, Observed ASV, Shannon, and Simpson metrics in QIIME 2. Beta diversity was assessed based on UniFrac distance matrices, and non-metric multidimensional scaling (NMDS) was performed to visualize differences in microbial community composition among samples. Community composition was characterized at the phylum and genus levels.

### 2.5. Statistical Analysis

All data were analyzed using IBM SPSS Statistics 27.0. One-way analysis of variance (ANOVA), followed by Tukey’s post hoc test for multiple comparisons, was performed for all data except the microbial abundance data. Because the microbial abundance data did not satisfy the assumption of normality, the non-parametric Kruskal–Wallis rank-sum test was used to assess differences among groups. GraphPad Prism 8.0.2 software was used for data visualization. Results are presented as mean ± standard error. *p* < 0.05 indicates a significant difference, while *p* < 0.01 indicates a highly significant difference.

## 3. Results

### 3.1. Performance of Biofilters in TMA Removal

**Figure 3:**
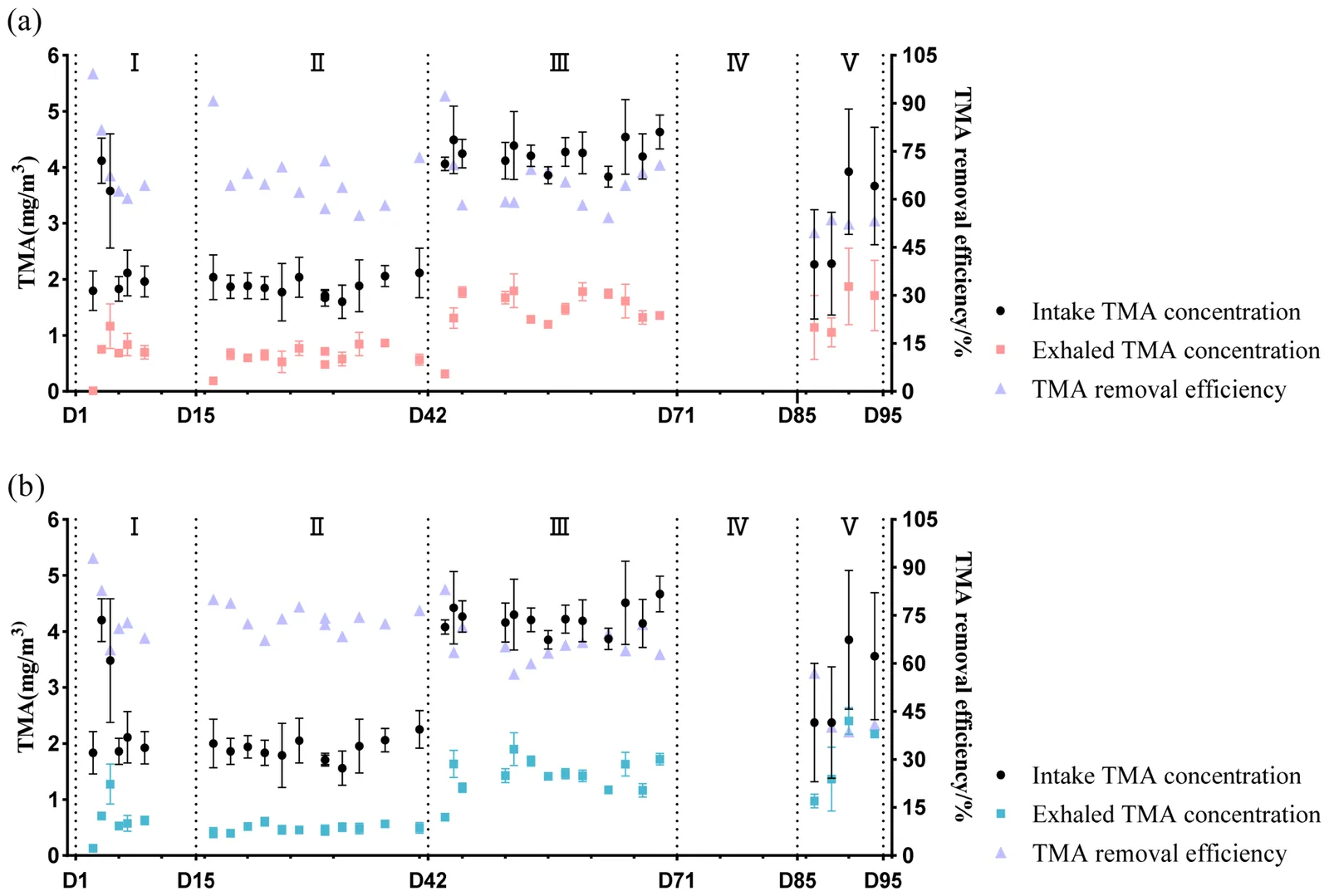

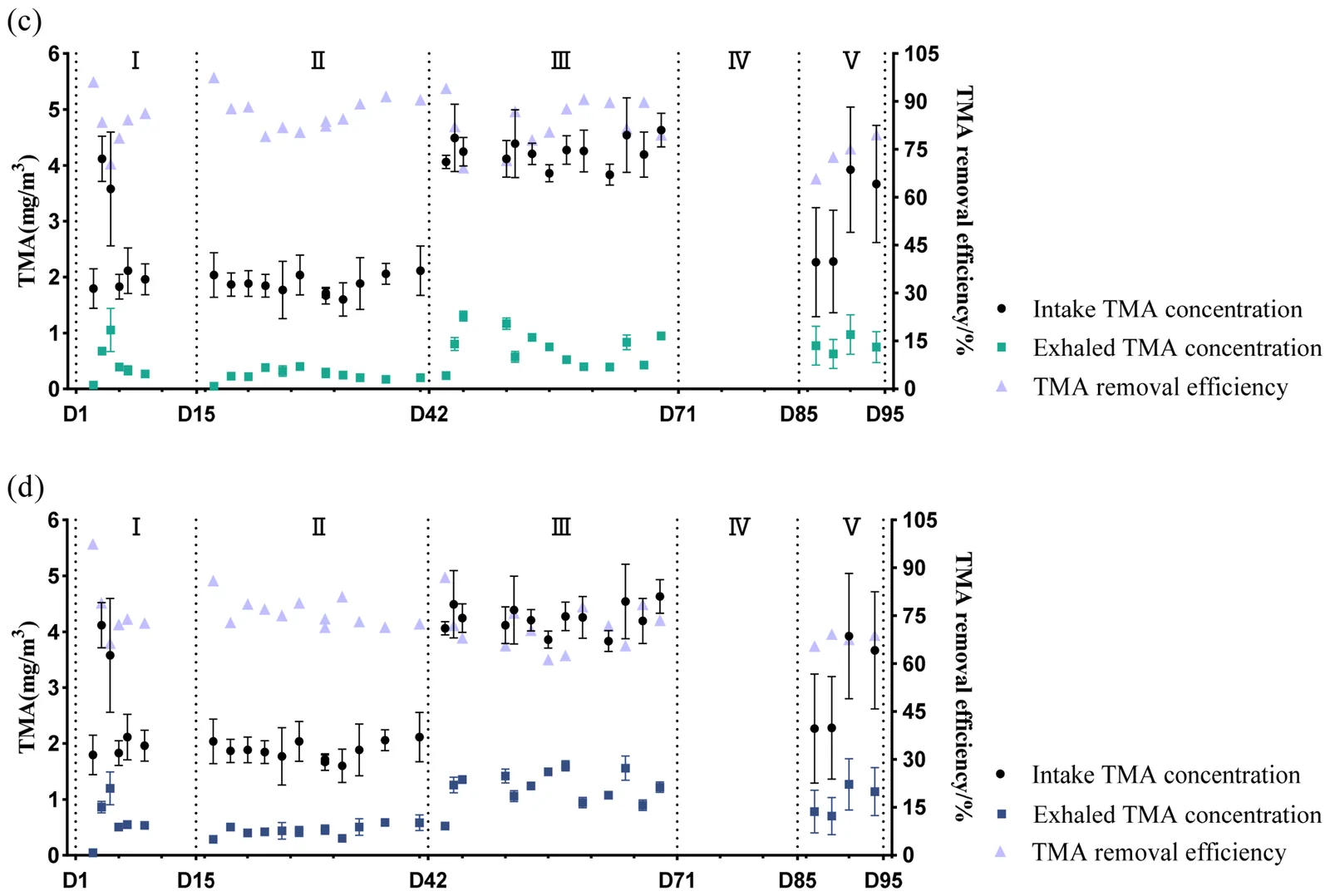
Measured TMA concentrations at the air inlet and outlet and calculated TMA removal efficiency for the four tested biofilters: (**a**–**d**) are for biofilters BF_A, BF_B, BF_C, and BF_D, respectively. The Roman numerals I, II, III, IV, and V indicate the five phases of the experiment, respectively.

During Phase I, TMA was not detected in the effluent during the first three days of operation (Figure 3). Thereafter, measurable TMA concentrations were observed at the outlets of all four biofilters, and removal performance progressively stabilized during the initial acclimation period. At the transitions to Phases II and III, transient increases in outlet TMA concentration were observed following changes in the operating conditions. During Phase III, the removal efficiencies of BF_A, BF_B, BF_C, and BF_D generally remained within approximately 55–70%, 60–70%, 75–90%, and 60–75%, respectively. Following the two-week shutdown period, BF_C retained the highest mean removal efficiency during Phase IV (73.33%), followed by BF_D (67.81%), BF_A (52.27%), and BF_B (44.21%). These results indicate that BF_C maintained the highest observed TMA removal performance across the tested operating periods.

### 3.2. Other Indicators of Biofilter Behavior

#### 3.2.1. Carbon Dioxide

**Figure 4:**
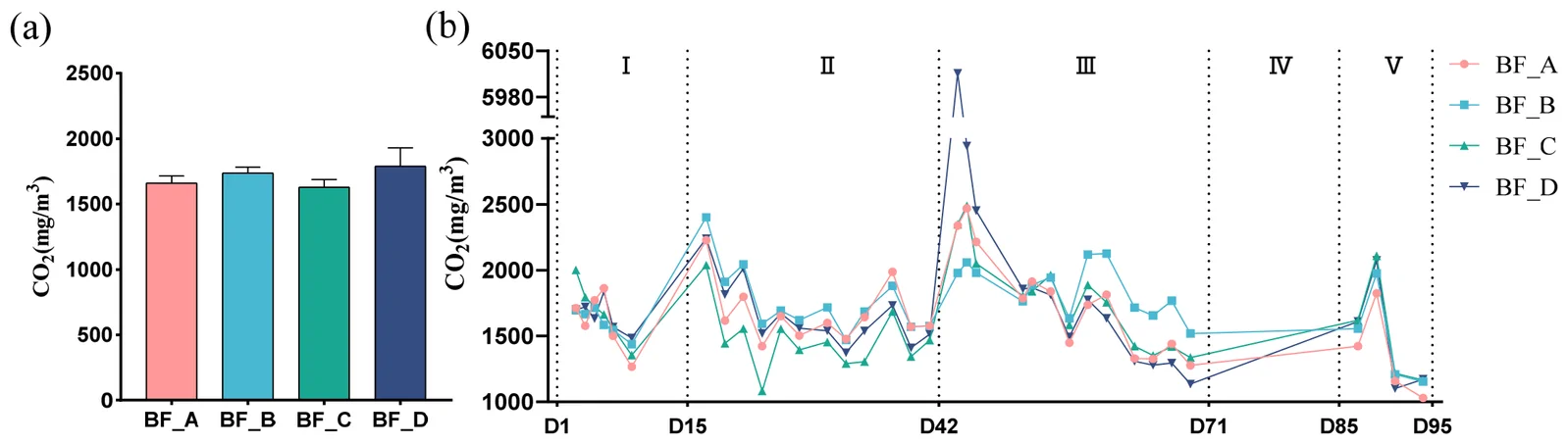
CO_2_ concentration measured at the biofilter outlet. (**a**) average, (**b**) variation during test period. The Roman numerals I, II, III, IV, and V indicate the five phases of the experiment, respectively.

Carbon dioxide is a main byproduct of biofiltration for removing air pollutants. The CO_2_ concentrations measured at the biofilter outlets were about 4 four times the ambient level (Figure 4a), with BF_B having a slightly higher level than the others, while BF_A exhibited the minimal fluctuation in CO_2_ concentration. Notably, the average CO_2_ concentration of BF_D showed an abrupt and atypical increase at the onset of Phase III (Figure 4b).

#### 3.2.2. Ammonia

**Figure 5:**
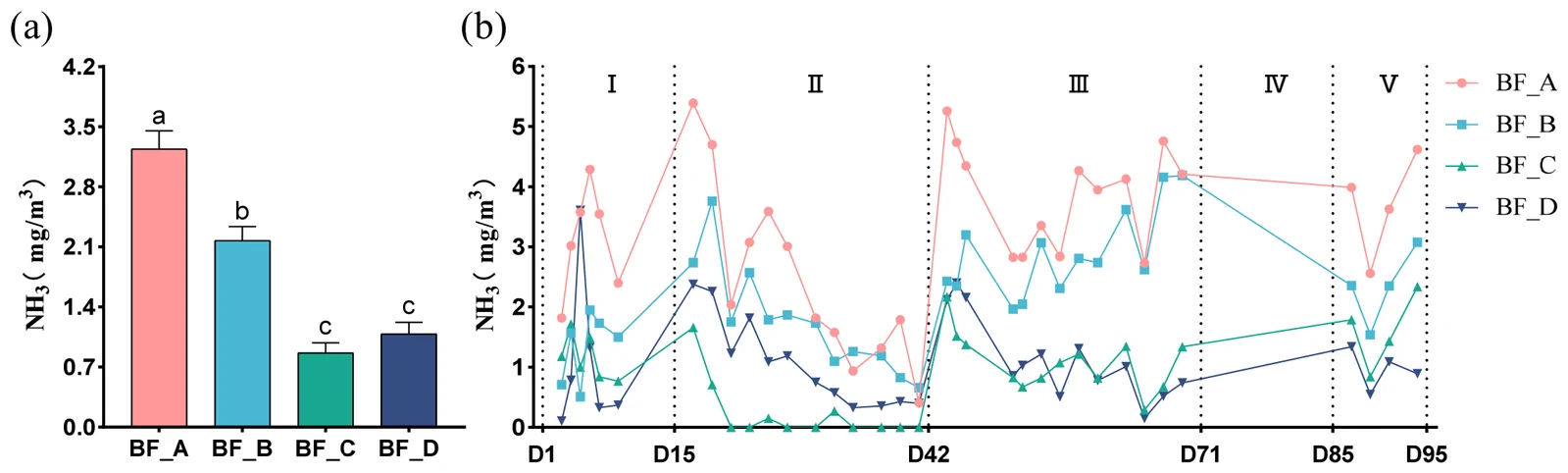
NH_3_ concentration measured at the biofilter outlet. (**a**) average and (**b**) variation during test period. The Roman numerals I, II, III, IV, and V indicate the five phases of the experiment, respectively. With different lowercase letters indicate significant differences (*p* < 0.05), and the same lowercase letters indicate no significant differences (*p* > 0.05).

Under aerobic conditions, microorganisms may convert TMA to DMA, then MMA, and finally to NH_3_. This means that NH_3_ could be a secondary pollutant in biofiltration treatment of TMA. The average NH_3_ concentrations at the outlets of four biofilters during the entire stable operation phase ranked in descending order as 3.2 mg/m^3^ for BF_A, 2.35 mg/m^3^ for BF_B, 1.1 mg/m^3^ for BF_D, and 0.69 mg/m^3^ for BF_C. Statistically, the NH_3_ of BF_A and BF_B were significantly higher than those of BF_C and BF_D (*p* < 0.05). In Phase II, the NH_3_ concentrations of BF_A, BF_B, and BF_D immediately reached a peak and then gradually declined until the end of this phase (Figure 5b). It is worthwhile to note that BF_C emitted very little NH_3_ in Phase II (the NH_3_ emission concentration remained near 0 mg/m^3^ after a small peak at the beginning). Throughout the entire operation period, BF_A exhibited the highest NH_3_ concentration (Figure 5b).

#### 3.2.3. Ammonium Nitrogen

**Figure 6:**
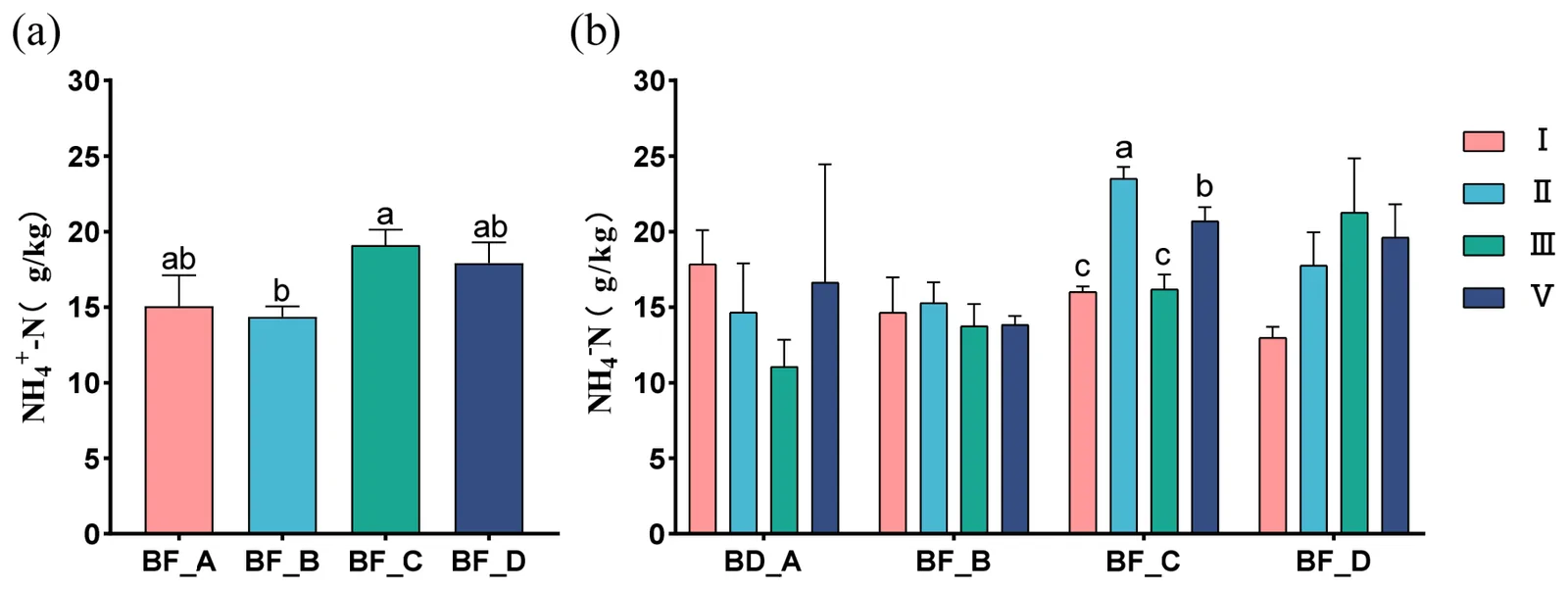
Measured NH_4_^+^-N content in filter media. (**a**) overall average, (**b**) average in each test phase. The Roman numerals I, II, III, and V indicate the four phases of the experiment, respectively. With different lowercase letters indicate significant differences (*p* < 0.05), and the same lowercase letters indicate no significant differences (*p* > 0.05).

Ammonium is a byproduct of enzyme oxidation of TMA under aerobic conditions. Statistically, the NH_4_^+^-N content in BF_B was significantly lower than that in BF_C (*p* < 0.05) (Figure 6). Furthermore, the NH_4_^+^-N content in the filter medium was significantly higher in BF_C than that in BF_B (*p* < 0.05), whereas no significant differences were detected between BF_A, BF_B, and BF_D (*p* > 0.05). This indicated that Nitrogen was mostly trapped in the filter medium instead of being emitted as ammonia in BF_C.

#### 3.2.4. Total Nitrogen Content (TN)

**Figure 7:**
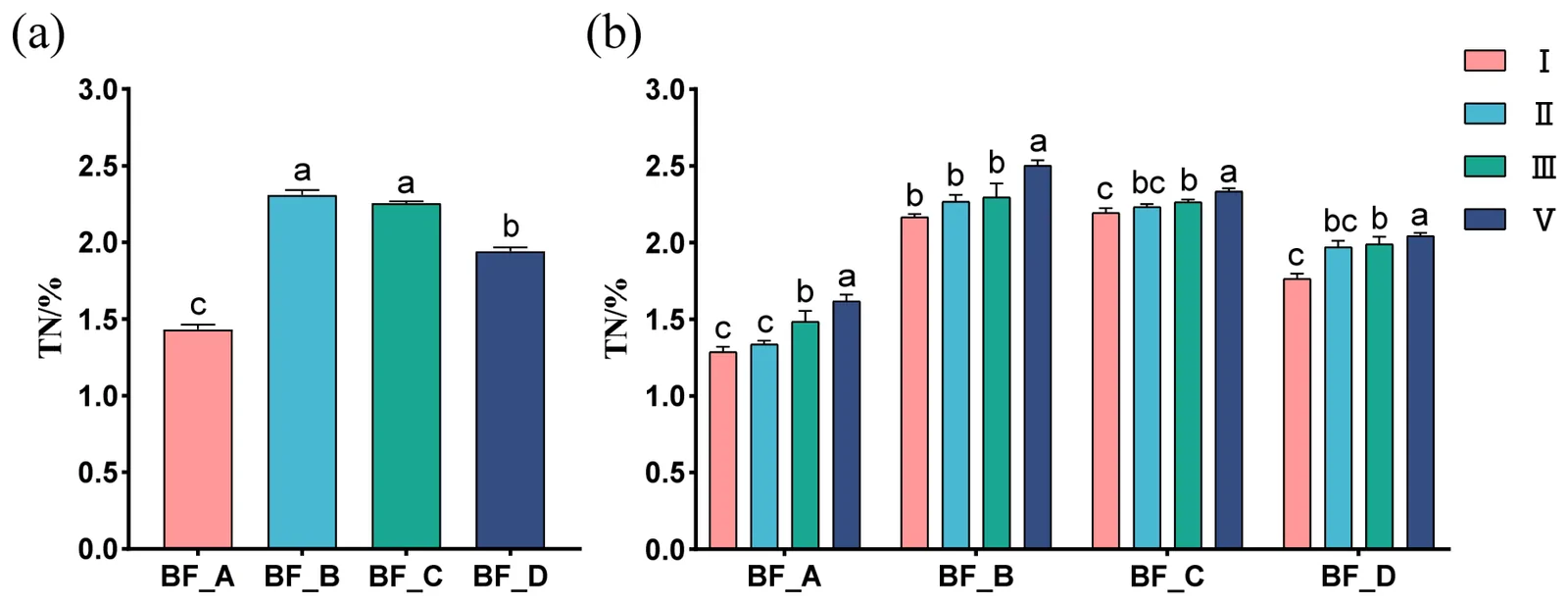
Measured total nitrogen content in filter media. (**a**) overall average, (**b**) average in each test phase. The Roman numerals I, II, III, and V indicate the four phases of the experiment, respectively. With different lowercase letters indicate significant differences (*p* < 0.05), and the same lowercase letters indicate no significant differences (*p* > 0.05).

The TN content in the biofilter media of BF_B and BF_C was significantly higher than that in BF_A and BF_D (*p* < 0.05) (Figure 7a). The overall average TN content in all biofilters was significantly higher in Phases II and III than in other phases (*p* < 0.05) (Figure 7a,b). The TN content in BF_A was significantly higher in Phases III and V than in Phases I and II. For BF_B, the TN content in Phase V was significantly higher than in all preceding phases. Similarly, the TN content in both BF_C and BF_D during Phase V was significantly higher than that in Phase I (*p* < 0.05).

### 3.3. Microbial Communities in the Biofiltration System

Among the four tested biofilters, BF_C exhibited the highest TMA removal efficiency (Section 3.2). Therefore, microbial analysis was performed on the filter medium samples from BF_C to investigate the structural characteristics of its microbial community at different time points, aiming to elucidate the patterns of its dynamic succession.

#### 3.3.1. Analysis of Microbial Diversity in Filter Media

**Figure 8:**
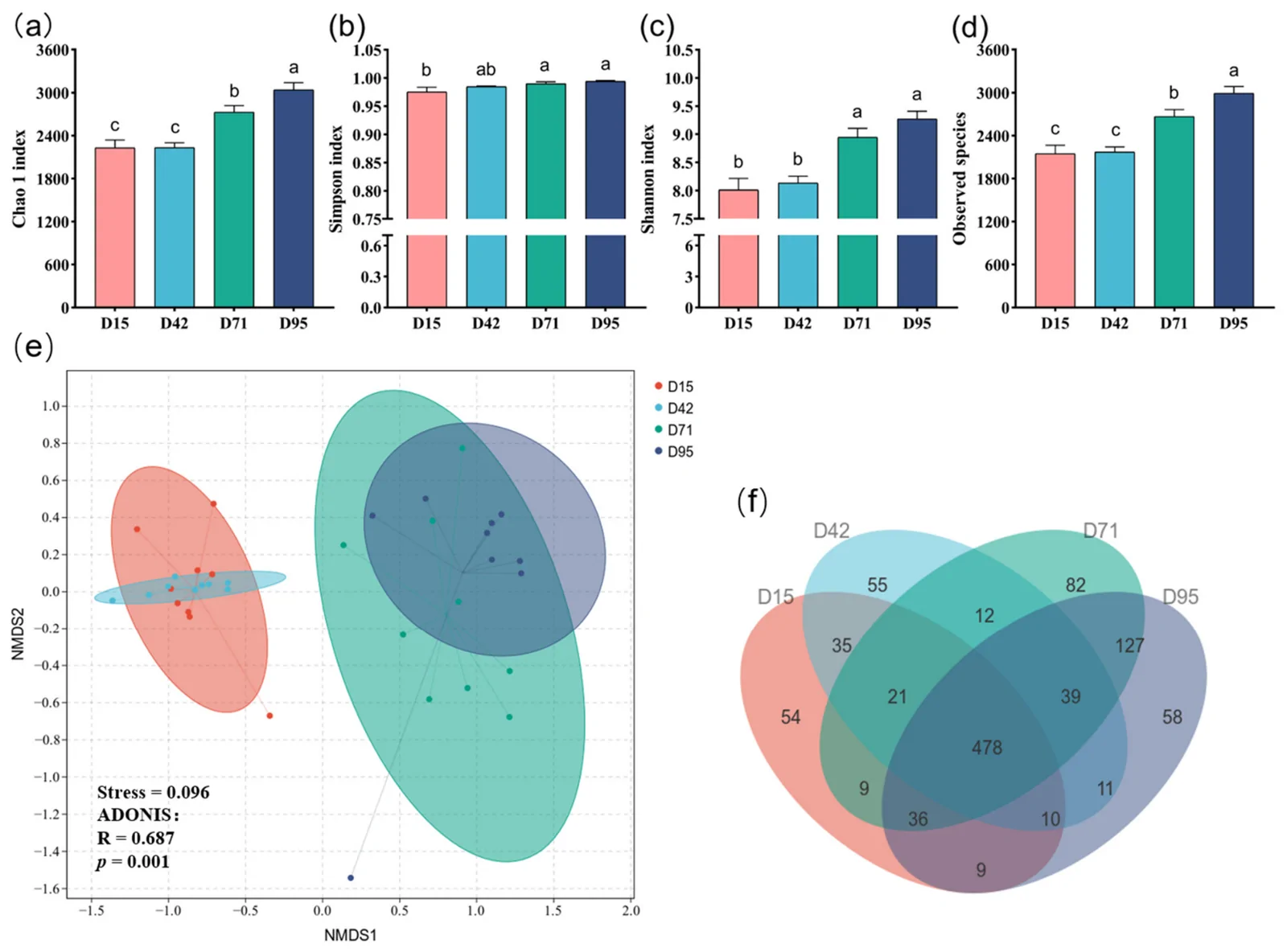
Diversity of microorganisms in biofilter media at different time periods. (**a**). Chao index of microbial α diversity of filter media; (**b**). Simpson index of microbial α diversity of filter media; (**c**). Shannon index of microbial α diversity of filter media; (**d**). Observed-species index of microbial α-diversity in filter media; (**e**). NMDS ordination of ASV-based microbial community composition based on UniFrac distances; (**f**). Genus-level Venn diagram showing the shared and stage-specific genera in filter media samples collected at D15, D42, D71, and D95; lowercase letters with different shoulder marks indicate significant differences (*p* < 0.05), and the same letters with the same labels indicate no significant differences (*p* > 0.05).

The α-diversity indices of the filter media microbiota, including the Chao1 index, Simpson index, Shannon index, and Observed species, showed significant increases following the experiment (Figure 8a–d). NMDS ordination based on UniFrac distances revealed temporal variation in the microbial community composition of the filter media across the four sampling stages. Permutational multivariate analysis of variance using the Adonis method further indicated significant differences in microbial community composition among the sampling stages (*p* = 0.001; Figure 8e). The genus-level Venn diagram showed that 54, 55, 82, and 58 genera were unique to D15, D42, D71, and D95, respectively, while 478 genera were shared across all four groups (Figure 8f).

#### 3.3.2. Microbial Community Composition at the Phylum and Genus Levels

**Figure 9:**
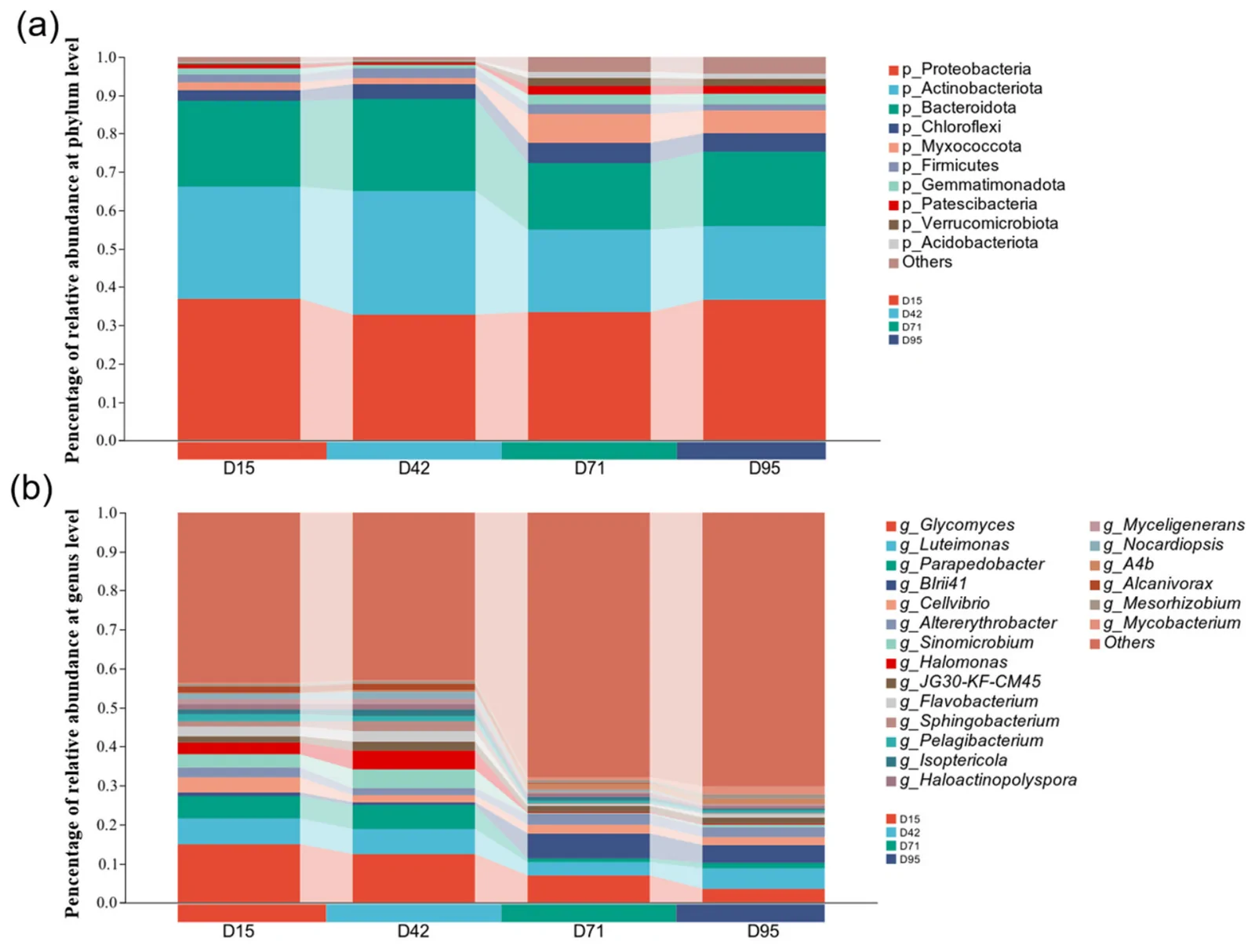
Microbial species composition at the phylum level (**a**) and the genus level (**b**) in biofilter media at different time points.

The microbial community composition at the phylum level is presented in Figure 9a. Seven dominant phyla, namely Proteobacteria, Actinobacteria, Bacteroidota, Chloroflexi, Myxococcota, Firmicutes, and Gemmatimonadota, collectively accounted for more than 90% of the relative abundance at each sampling time point. Proteobacteria, Actinobacteria, and Bacteroidetes were the predominant phyla in the filter media. The relative abundance of Proteobacteria decreased from 36.82% at D15 to 32.84% at D42, before recovering to 33.41% at D71 and 36.69% at D95. In contrast, Actinobacteria increased from 29.41% at D15 to 32.16% at D42, followed by a decline to 21.73% at D71 and 19.26% at D95. Bacteroidota exhibited a similar pattern, increasing from 22.34% at D15 to 24.01% at D42, decreasing to 17.27% at D71, and recovering slightly to 19.49% at D95. The combined relative abundance of these three phyla decreased from 88.57% and 89.01% at D15 and D42, respectively, to 72.41% at D71, before increasing to 75.44% at D95. The other four dominant phyla showed distinct temporal patterns. Chloroflexi increased from 2.79% at D15 to 5.31% at D71, followed by a slight decrease to 4.85% at D95. Myxococcota decreased from 2.06% at D15 to 1.53% at D42, then increased to 7.57% at D71 and remained relatively abundant at 5.80% at D95. Firmicutes showed comparatively limited variation, with relative abundances of 1.97%, 2.54%, 2.57%, and 1.77% at D15, D42, D71, and D95, respectively. Gemmatimonadota accounted for 1.74%, 0.89%, 2.43%, and 2.64% at the corresponding time points. Overall, the phylum-level profiles indicated temporal changes in the relative representation of dominant bacterial groups, particularly after D42.

The relative abundance profiles of the 20 most abundant genera in the filter medium at the four sampling time points are shown in Figure 9b. At D15, the genera with relative abundances exceeding 2% were *Glycomyces* (17.22%), *Luteimonas* (7.70%), *Parapedobacter* (6.50%), *Cellvibrio* (4.57%), *Sinomicrobium* (4.04%), *Halomonas* (3.44%), *Flavobacterium* (2.85%), and *Altererythrobacter* (2.80%). At D42, genera with relative abundances greater than 2% included *Glycomyces* (14.59%), *Parapedobacter* (7.45%), *Luteimonas* (7.34%), *Sinomicrobium* (5.53%), *Halomonas* (5.48%), *Flavobacterium* (3.09%), *Sphingobacterium* (2.89%), *JG30-KF-CM45* (2.82%), *Altererythrobacter* (2.35%), *Cellvibrio* (2.13%), *Nocardiopsis* (2.04%), and *Alcanivorax* (2.04%). At D71, genera with relative abundances greater than 2% included *Glycomyces* (8.56%), *BIrii41* (8.12%), *Luteimonas* (4.35%), *Altererythrobacter* (3.33%), *Cellvibrio* (2.90%), *A4b* (2.06%), and *JG30-KF-CM45* (2.02%). At D95, genera with relative abundances greater than 2% included *Luteimonas* (6.64%), *BIrii41* (5.64%), *Glycomyces* (4.24%), *Altererythrobacter* (3.03%), *Cellvibrio* (2.69%), and *Mycobacterium* (2.61%). Temporal changes were evident in the relative abundances of several dominant genera. *Glycomyces* was the most abundant genus at D15, D42, and D71, but its relative abundance progressively decreased from 17.22% to 4.24% over the monitoring period. In contrast, *Luteimonas* remained among the predominant genera at all four time points, with its relative abundance decreasing from 7.70% at D15 to 4.35% at D71 before recovering to 6.64% at D95. *BIrii41* was among the dominant genera at D71 and D95, accounting for 8.12% and 5.64%, respectively, and ranked above *Glycomyces* at D95. *Parapedobacter*, *Sinomicrobium*, and *Halomonas* were relatively abundant at D15 and D42 but were no longer among the genera exceeding 2% at D71 and D95. By comparison, *Altererythrobacter* maintained a relatively consistent proportion, ranging from 2.35% to 3.33%, while *Cellvibrio* remained above 2% at all four time points despite fluctuations in its relative abundance. These patterns indicate that the genus-level community composition changed over time, with a gradual reduction in the relative representation of *Glycomyces* and a greater contribution from other genera, including *Luteimonas* and *BIrii41*, toward the end of the monitoring period.

#### 3.3.3. Differential Abundance Analysis of Microbial Taxa in the Filter Medium

**Figure 10:**
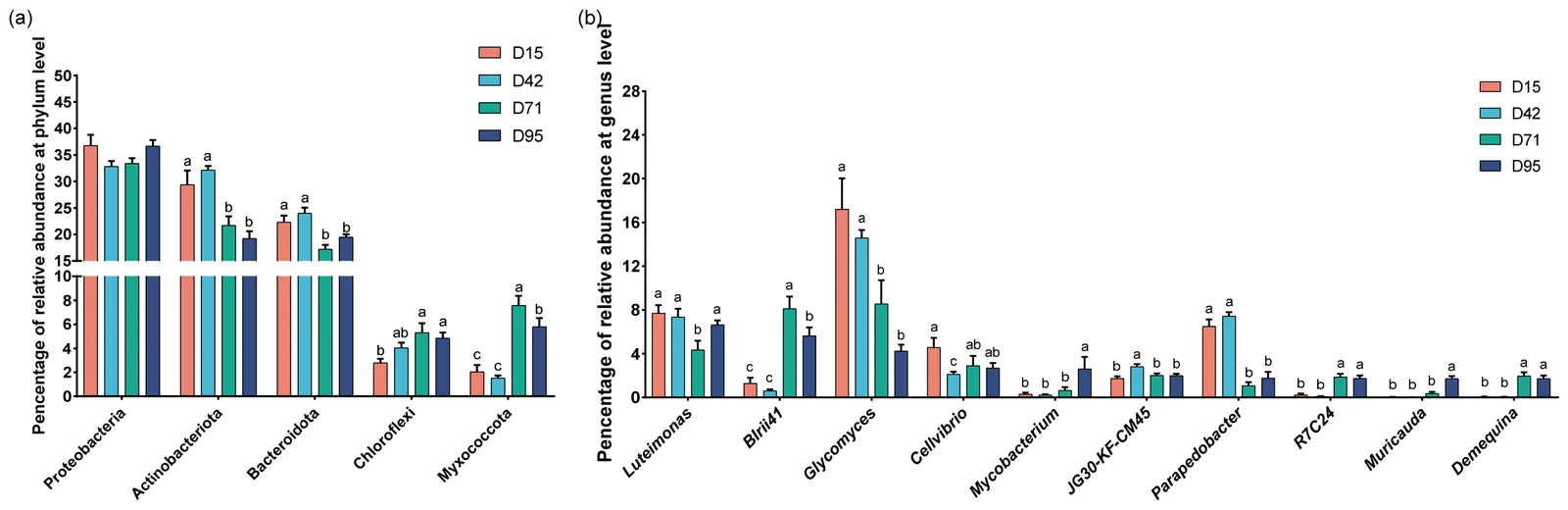
Differential species in filter media at different time periods. Note: (**a**). Species with significant differences in the relative abundance of filter media microorganisms at the phylum level at different time periods; (**b**). Species with significant differences in the relative abundance of filter media microorganisms at the genus level at different time periods. With the lowercase letters of different shoulder marks indicated significant differences (*p* < 0.05), and the same letters of the same labels indicated no significant differences (*p* > 0.05).

The relative abundances of the top 5 microbial taxa exhibiting significant differences at the phylum level across sampling periods are presented in Figure 10a. The relative abundances of Bacteroidetes and Actinobacteria were significantly lower at D71 and D95 compared with those at D15 and D42 (*p* < 0.05). In contrast, the abundances of Myxococcota and Chloroflexi were significantly higher at D71 and D95 than at D15 and D42 (*p* < 0.05).

At the genus level, significant temporal shifts were observed (Figure 10b). The relative abundances of *Biri41*, *Mycobacterium*, *R7C24*, *Muricauda*, and *Demequina* were significantly higher at D71 and D95 than at D15 and D42, whereas the abundances of *Glycomyces* and *Parpedobacter* were markedly reduced (*p* < 0.05). In contrast, the abundance of *Cellvibrio* was significantly lower at D42 than at all other time points. The abundance of *JG30-KF-CM45* was uniquely elevated at D42. The abundance of *Luteimonas* was significantly reduced specifically at D71 compared to D15, D42, and D95. Finally, the abundances of *Lyucobacterium* and *Muricauda* were significantly higher at D95 than at D15, D42, and D71 (*p* < 0.05).

### 3.4. Correlation Analysis Between Filter Media Microorganisms and TMA Removal Efficiency

**Figure 11:**
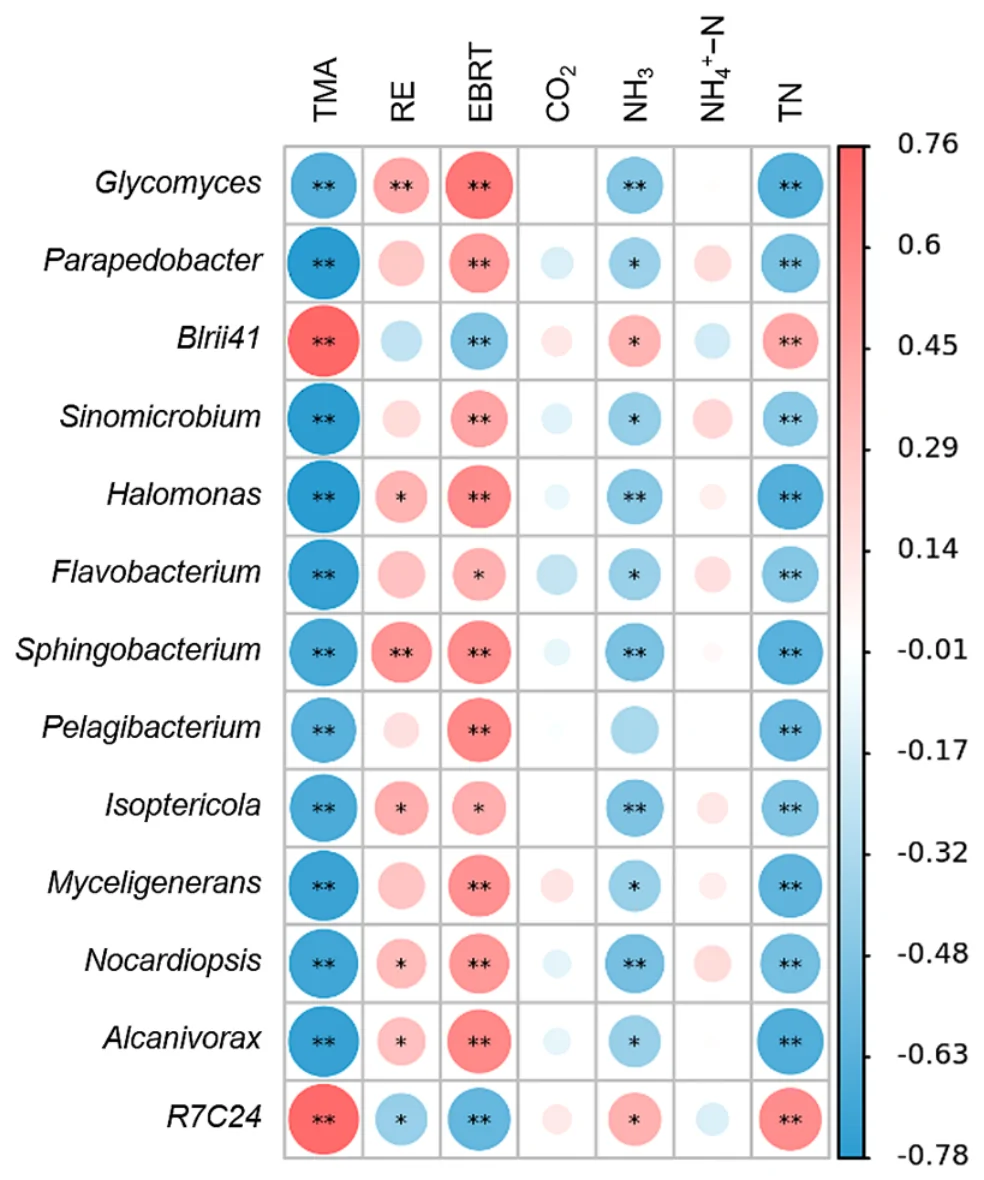
Correlation analysis of microbial species in filter media with biofilter behavior indicators. Circle size indicates the strength of the correlation, with larger circles representing stronger correlations and smaller circles representing weaker correlations. * *p* < 0.05, ** *p* < 0.01.

Spearman correlation analysis was conducted to evaluate the relationships between microbial genera in the filter media and the measured biofilter behavior indicators (Figure 11). The results indicated that *Blrii41* and *R7C24* exhibited significant negative correlations with TMA inlet concentration (*p* < 0.01), as well as NH_3_ emissions and TN content (*p* < 0.05). However, neither genus displayed a significant correlation with TMA removal efficiency (*p* > 0.05). Genera such as *Glycomyces*, *Parapedobacter*, *Sinomicrobium*, and *Halomonas* showed significant negative correlations with TMA inlet concentrations. Notably, *Glycomyces*, *Sphingobacterium*, and *Halomonas* exhibited highly significant positive correlations with TMA removal rates (*p* < 0.01).

## 4. Discussion

### 4.1. Filter Media

Composite biofilter media are commonly prepared by combining compost with bulking agents such as sawdust or perlite, and previous studies have reported their effectiveness in removing various volatile organic compounds (VOCs) [24,25,26]. In the present study, mature compost and recycled cattle-manure-based bedding were selected as the principal organic substrates, whereas sawdust and crushed corn cobs were incorporated as lignocellulosic bulking agents. Mature compost was included as a partially decomposed organic matrix that could provide nutrients and an initial microbial inoculum, whereas cattle manure bedding was evaluated as an alternative manure-derived material. Sawdust and crushed corn cobs were used to formulate composite media with the potential to maintain bed structure and facilitate gas distribution. The four formulations were designed to compare different combinations of these materials rather than to establish an optimized mixing ratio. Specifically, media A and C shared the same volumetric ratio of 10:9:1 but differed in their principal substrate: cattle manure bedding was used in media A, whereas mature compost was used in media C. Media B combined mature compost and cattle manure bedding at a 1:1 ratio, whereas media D contained mature compost, cattle manure bedding, sawdust, and crushed corn cobs at a ratio of 6:6:7:1.

Among the four formulations, BF_C exhibited the highest TMA removal efficiency, whereas BF_D showed intermediate performance and BF_A and BF_B showed lower removal efficiencies. The superior performance of BF_C suggests that combining mature compost with sawdust and crushed corn cobs was suitable for TMA removal under the operating conditions tested. This observed discrepancy may be tentatively explained by two potential contributing factors. First, the relatively low porosity of cattle manure bedding likely resulted in reduced air permeability in BF_B compared with BF_C. Second, the organic carbon content provided by cattle manure bedding was presumably lower than that supplied by sawdust and corn cobs [27]. Previous studies have shown that the total carbon content of cattle manure bedding decreases progressively during processing [28]. This tentative inference aligns with the comparison between Media A and Media C: although both contained sawdust and crushed corn cobs, Media A was formulated with cattle manure bedding, whereas Media C was based on mature compost. The significant difference in removal efficiency between the two media might be attributed to two primary factors. Firstly, during the composting process, the organic matter in mature compost undergoes partial mineralization, resulting in a substrate that is more readily assimilable by microorganisms. In contrast, cattle manure bedding typically remains in a raw state and contains a higher proportion of recalcitrant compounds, such as cellulose and hemicellulose, which can hinder the overall metabolic activity of the biofilter [29]. Secondly, mature compost harbors a rich and well-acclimated community of functional microorganisms [30]. Conversely, although the cattle manure bedding contains microorganisms, its microbial community structure was likely less developed, with lower abundances of key functional taxa, thereby necessitating a longer acclimation period to achieve stable treatment efficiency. Consistent with this, the removal efficiency of BF_B was significantly lower than that of BF_C during operation. This suggested that although cattle manure bedding was derived from manure, it contained fewer available nutrients and harbored a less diverse microbial community than mature compost. Media D, composed of mature compost, cattle manure bedding, sawdust, and crushed corn cobs (6:6:7:1 by volume), exhibited an intermediate removal efficiency, lower than that of BF_C but higher than those of BF_A and BF_B. This suggested that cattle manure bedding was less effective than mature compost as a nutrient source and also inferior to sawdust as a bulking agent for maintaining adequate porosity.

### 4.2. Ammonium Nitrogen and Total Nitrogen

Previous research has established that bacterial metabolism of TMA proceeds via two primary pathways. Under aerobic conditions, TMA is sequentially oxidized by enzymes such as TMA monooxygenase, TMA oxidase, TMA demethylase, and methylamine monooxygenase, generating TMA N-oxide, dimethylamine, and methylamine as intermediates, which are ultimately converted to formaldehyde and ammonium. Alternatively, under anaerobic conditions, TMA can be degraded through the action of TMA dehydrogenase and dimethylamine dehydrogenase, yielding dimethylamine and methylamine as successive products [15,31]. During Phase I of the experiment, NH_3_ was detected in the outlet gas of the biofilters, prompting its inclusion as a key monitoring parameter, along with subsequent measurements of NH_4_^+^-N and TN in the collected media samples. The results showed that the NH_4_^+^-N content in the media of BF_B was significantly lower than that in BF_C. In contrast, NH_3_ emissions from BF_A and BF_B were substantially higher than those observed in BF_C and BF_D. As a major form of nitrogen loss from the filter media, NH_4_^+^-N is strongly influenced by temperature, pH, and the activity of ammonia-oxidizing bacteria. Under lower pH conditions, nitrogen predominantly remains in the non-volatile NH_4_^+^-N form, whereas under higher pH, a considerable fraction of NH_4_^+^-N can shift to gaseous NH_3_ and be lost through volatilization [32]. This observed pattern could be plausibly explained by a gradual rise in pH within Media A and B over the operating period, which would in turn promote the conversion of NH_4_^+^-N into volatile NH_3_. By contrast, it may be postulated that Media C and D possessed greater buffering capacity and more stable pH conditions, which would limit nitrogen loss through volatilization. Although microbial assimilation of TMA-derived nitrogen may have contributed to this retention, the relative contributions of microbial assimilation, ammonium retention, and other nitrogen pools could not be separately quantified in the present study. Collectively, these results demonstrated that using mature compost as a primary filter medium not only enabled the recycling of a dairy manure by-product but also promoted nutrient recovery during gas purification. As a result, the compost became enriched with nitrogen, enhancing its agronomic value for subsequent agricultural applications.

### 4.3. Microbial Community

The performance of biofilters, as an efficient and low-cost technology for malodor treatment, is largely governed by the metabolic activities and functional capabilities of the resident microbial communities within the filter media [33,34]. Consequently, understanding the structural characteristics and temporal dynamics of these communities is essential for elucidating the microbiological mechanisms underlying the efficient removal of odorous gases [35,36]. The α-diversity of the microbial community provides information on community richness and evenness and can be useful for characterizing microbial community development during biofilter operation [37]. The results showed significant increases in the Chao1 and Observed species indices, indicating the successful introduction and colonization of new taxa during biofilter operation. Concurrent increases in the Shannon and Simpson diversity indices suggested a more diverse microbial community, potentially reflecting a more even distribution of relative abundances among taxa. NMDS ordination based on UniFrac distances revealed temporal variation in the microbial community structure of the filter media, and the genus-level Venn diagram further showed that 478 genera were shared across D15, D42, D71, and D95, whereas 54, 55, 82, and 58 genera were unique to each sampling stage, respectively. These findings suggest that, although a substantial proportion of the microbial community was maintained throughout biofilter operation, stage-specific differences in genus-level composition also emerged over time. This pattern is consistent with temporal microbial succession during long-term operation, potentially reflecting changes in the environmental conditions within the filter media.

Analysis of 16S rRNA gene sequencing data revealed that the microbial communities within the filter media were predominantly composed of the phyla Proteobacteria, Actinobacteriota, Bacteroidota, and Chloroflexi. The dominance of these phyla was consistent with observations reported in previous studies of comparable biofiltration systems, highlighting their widespread adaptability and functional importance in biofilter environments [38,39]. These bacterial phyla consistently represented major components of the microbial community throughout the experimental period, although their relative abundances varied over time. Notably, Proteobacteria and Actinobacteria are widely recognized as key contributors to fundamental ecological processes, including nitrogen cycling and organic matter decomposition. As one of the most ubiquitous and metabolically versatile bacterial phyla, Proteobacteria encompass taxa capable of diverse biochemical functions, such as the degradation of organic pollutants, nitrification and denitrification, and carbon metabolism, thereby conferring strong functional resilience to the biofilter microbial community [40,41]. Actinobacteria are key functional microorganisms in natural and engineered ecosystems, renowned for producing a wide array of extracellular enzymes capable of degrading complex and recalcitrant organic substances, such as cellulose and lignin. Through these activities, they contribute substantially to organic matter turnover, nutrient cycling, and the enhancement of soil fertility, while also playing an important role in the biodegradation and detoxification of organic pollutants. In the present study, Proteobacteria consistently remained the dominant phylum throughout the entire operational period, exhibiting no significant fluctuations in relative abundance. This apparent stability was likely attributable to the exceptionally high taxonomic and functional diversity within the Proteobacteria phylum, which encompasses numerous species with broad metabolic versatility. Such metabolic plasticity enables Proteobacteria to adapt to fluctuating environmental conditions and withstand various stressors, including changes in substrate availability and exposure to diverse odorous compounds [42,43]. Contrary to observations reported in some previous studies, the relative abundance of Actinobacteria declined significantly on D71 and D95 compared with D15 and D42. This decrease may reflect the progressive depletion of readily biodegradable organic substrates in the filter media as the biofilter matured and labile carbon sources were gradually consumed [44]. In contrast, the relative abundances of Myxococcota, Chloroflexi, Patescibacteria, Verrucomicrobiota, Acidobacteriota, and Planctomycetota increased significantly over the course of the experiment, a trend consistent with findings reported by Ni et al. [45,46]. Functionally, members of the phylum Chloroflexi have been shown to participate in nitrate reduction processes using organic carbon as an electron donor. Meanwhile, Acidobacteriota and Verrucomicrobiota encompass taxa capable of organic matter decomposition, carbon acquisition, nitrogen fixation, and phosphorus solubilization. Collectively, the temporal enrichment of these taxa indicates substantial restructuring of the microbial community during long-term operation. Their potential contributions to TMA removal remain to be experimentally verified.

The dominant bacterial genera detected in the filter media included *Luteimonas*, *BIrii41*, *Glycomyces*, *Altererythrobacter*, *Cellvibrio*, and *Mycobacterium*. Among these, *Luteimonas* has been frequently reported in composting and biofiltration systems, where it plays an important role in the degradation of organic matter and actively participates in nitrogen transformation and cycling processes [47,48,49]. In the present study, temporal changes in the abundance of *Luteimonas* occurred alongside changes in NH_3_ emissions, NH_4_^+^-N content, and TN levels; however, the present data do not establish a direct functional relationship between this genus and nitrogen transformation. Notably, the genus *BIrii41*, belonging to the phylum *Proteobacteria*, exhibited a significant positive correlation with the nitrogen content of the filter media. The relative abundance of *BIrii41* was below 2% during the initial phases but rose to approximately 8% in the stable operation phase (Phase III) and remained around 7% at the end of the experiment. This pronounced increase, coupled with its sustained presence, indicated that *BIrii41* possessed a remarkable adaptability to the TMA-loaded environment. Correlation analysis indicated that *BIrii41* displayed a significant negative correlation with the inlet TMA concentration, but no significant correlation was observed with TMA removal efficiency. Furthermore, the relative abundance of *Glycomyces* gradually decreased as the experiment progressed. Although *Glycomyces* showed a positive correlation with TMA removal efficiency, its relative abundance decreased over the course of operation. This finding suggests an association between the temporal dynamics of *Glycomyces* and biofilter performance, whereas its specific role and underlying mechanism remain to be elucidated.

## 5. Conclusions

Among the four tested packing configurations, BF_C, consisting of mature compost, wood chips, and crushed corn cobs at a volume ratio of 10:9:1, showed the highest observed TMA removal performance, with removal efficiencies generally exceeding 80% during stable operation. TMA removal was accompanied by ammonia emission and nitrogen accumulation in the filter media, indicating nitrogen transformation and retention during biofiltration. In BF_C, microbial richness and diversity increased over the operation period, accompanied by substantial community succession, and several bacterial genera were associated with TMA removal performance; however, their specific roles in TMA degradation require further investigation.

## Figures and Tables

**Table 1:** Biofilter operational parameters during each phase of the experiment.

Days	Operational Stage	Gas Flow (L/s)	EBRT/s	Inlet TMA Concentration (mg/m^3^)
D1–D14	Phase I	0.197 ± 0.017	57.15 ± 5.86	2.57 ± 0.41
D15–D42	Phase II	0.253 ± 0.008	42.70 ± 1.25	1.88 ± 0.05
D43–D71	Phase III	0.286 ± 0.036	28.02 ± 1.37	4.23 ± 0.07
D72–D85	Phase IV	/	/	/
D86–D95	Phase V	0.351 ± 0.05	30.23 ± 0.40	3.04 ± 0.44

Note: Phase I is the initialization period; Phase II and III are the stable operation periods; Phase IV is the shutdown period; Phase V is the resumption period.

## Data Availability

The original contributions presented in the study are included in the article; further inquiries can be directed to the corresponding authors.
